# The other-race effect in 3-year-old German and Cameroonian children

**DOI:** 10.3389/fpsyg.2014.00198

**Published:** 2014-03-18

**Authors:** Janina Suhrke, Claudia Freitag, Bettina Lamm, Johanna Teiser, Ina Fassbender, Sonja Poloczek, Manuel Teubert, Isabel A. Vöhringer, Heidi Keller, Monika Knopf, Arnold Lohaus, Gudrun Schwarzer

**Affiliations:** ^1^Department of Developmental Psychology, Justus-Liebig-University GiessenGiessen, Germany; ^2^University of OsnabrueckOsnabrueck, Germany; ^3^University of BielefeldBielefeld, Germany; ^4^Goethe-University Frankfurt am Main, Frankfurt am MainGermany

**Keywords:** other-race effect, children, face recognition, Germany, Cameroon

## Abstract

Recognizing individual faces is an important human ability that highly depends on experience. This is reflected in the so called other-race effect; adults are better at recognizing faces from their own ethnic group, while very young infants do not show this specialization yet. Two experiments examined whether 3-year-old children from two different cultural backgrounds show the other-race effect. In Experiment 1, German children (*N* = 41) were presented with a forced choice paradigm where they were asked to recognize female Caucasian or African faces. In Experiment 2, 3-year-olds from Cameroon (*N* = 66) participated in a similar task using the same stimulus material. In both cultures the other-race effect was present; children were better at recognizing individual faces from their own ethnic group. In addition, German children performed at a higher overall level of accuracy than Cameroonians. The results are discussed in relation to cultural aspects in particular.

## INTRODUCTION

The other-race-effect (ORE) is a robust finding in adults’ face recognition, with more accurate discrimination and recognition of faces of one’s own race compared to faces from a different race (e.g., Meissner and Brigham, 2001). It is impressive that the ORE emerges very early in life between 3 and 9 months of age (Kelly et al., 2007). This holds true for faces of different races and for infants from different cultures suggesting the universality of the phenomenon (Kelly et al., 2009). From the infant results the question arises whether such a general phenomenon of face recognition as the ORE continues into early childhood. On the one hand a continuous transition seems plausible as the ORE is found in older children and adults, but on the other hand, studies on the ORE beyond infancy are rare and moreover provided inconsistent results between the ages of 3 to 8 years. Studying the transitions from infancy to early childhood always bears a methodological challenge, as usually different tasks are to be used at the different ages. Whereas looking time tasks are used to study infants’ face recognition, matching tasks are used beyond infancy. The youngest age group tested with a matching paradigm are 3-year olds in one study by Sangrigoli and de Schonen (2004). Among other age groups they investigated 3-year old Caucasian children’s face recognition using Caucasian and Asian faces. They found a floor effect and no ORE in the 3-year olds. However, after prolonging the presentation times of the faces they showed the ORE at that age. Such an influence of encoding duration on the emergence of the ORE suggests some kind of instability of the ORE in 3-year-olds which makes it important to further investigate the ORE at that age. Additionally, from a methodological perspective, an open question is whether an ORE in 3-year-old children can be observed when two ethnic groups are tested with the same face material. Showing the ORE in only one ethnic group, as by Sangrigoli and de Schonen (2004), cannot rule out that faces of one certain ethnic group simply contain more information and are therefore somewhat easier to distinguish than faces of the other ethnic group. Therefore, the present study aimed at further investigating the ORE in 3-year-old children by testing children from two different ethnic groups, Caucasian and African children from Germany and Cameroon, respectively, with a matching paradigm that provides the children with sufficient encoding times.

Previous research on the ORE in infants revealed that, within the first weeks of life, infants do not show a preference for faces of their own race, whereas at 3 months they do (Kelly et al., 2005; Bar-Haim et al., 2006). Within the first 9 months of life infants’ abilities to discriminate faces narrows down to faces from their own ethnicity associated with a decreasing ability to differentiate faces from different ethnic groups or even species (Kelly et al., 2007), supposedly based on differential experience with those faces. Based on the rather consistent evidence from infant and adult studies one could assume a fairly stable ORE present in children from 9 months on.

However, studies on the development in other perceptual-cognitive domains such as intuitive physics demonstrated that competencies from infancy are not shown to the same extent in early childhood. For example, Aschersleben et al. (2013) investigated the understanding of collision events in 3- to 5-year old children with a forced-choice task, which was based on the same stimuli that had been used in a looking time study with infants before. Whereas 10-month-old infants looked longer on inconsistent trials indicating an understanding of collision events no such understanding was found for the majority of the 3- to 4-year olds. One reason for the apparent incompetence of young children in domains where infants appeared to “know better’’ is seen in the different task demands in both age groups. Naturally, testing children in the first year of life requires different methods than doing so with older children.

Such a change in paradigm also takes place in the study of the ORE when looking time measures in the habituation-dishabituation or preferential looking paradigm are no longer age appropriate to test children beyond the first year of life. Up to now, there are no studies on the ORE beyond infancy until around the age of 3, when children can be tested with adaptations of adult measures of face recognition. The matching paradigm, where a once presented target face has to be recognized when paired with an unknown face, comes closest to the looking time tasks used with infants. Using such a paradigm Sangrigoli and de Schonen (2004) investigated the ORE in 3-, 4-, and 5-year-old Caucasian children with Caucasian and Asian faces. A target face was presented for 500 ms first, followed by a slide displaying the identical picture of the target face and a distractor face after a short interval of 1000 ms. Children were then asked to either point to the target face (for the 3-year-olds) or push an according button (4 years and older). The results showed the ORE for the 4- and 5-year-olds, but performance close to floor level in the 3-year olds. After extending the presentation times of the target from 500 to 1000 ms for the 3-year-olds, and thereby facilitating the task, they also showed an ORE at that age. The longer presentation times allowed for longer encoding of the target pictures and probably enabled the children to better memorize the faces. Interestingly, prolonging the presentation times only increased children’s own-race face recognition, other-race faces were still recognized at floor level with either presentation time. The results demonstrated that the ORE is found in Caucasian children as young as 3 years under the condition that children are given enough time to encode the target pictures. 

This provides evidence for the ORE in early childhood, yet, questions the stability of the effect and the impact of further methodological variables, such as characteristics of the stimulus material. Could the results of the worse recognition of other-race faces in Caucasian children be caused by the fact that these faces are less distinct and generally harder to distinguish than children’s own-race faces? Thus, left open is the question whether the ORE can be observed in 3-year-olds from different ethnic groups using the same face stimuli, being familiar (own-race) to one group and unfamiliar (other-race) to the other and vice versa. This would rule out alternative explanations for the results gained from studying only one culture. Moreover, investigating children from two cultures would allow showing that, as suggested by infant results, the ORE is indeed universal.

Looking at studies on the ORE in older children also does not provide clear conclusions for that age range, because those studies, too, yielded mixed results. Some studies with children from 5 years on found the ORE for all tested age groups. Pezdek et al. (2003) tested 5- and 8-year-olds as well as adults of African American or European American background and revealed an interaction of the race of the target face to be recognized and the race of the participant, thus indicating an ORE for each age group. De Heering et al. (2010) also found the ORE for Caucasian faces compared to Asian faces in Caucasian children from 6 years on. Other studies, in contrast, did not find the ORE for all tested age groups. For example, Chance et al. (1982) tested the recognition of Caucasian and Japanese faces in Caucasian 1st to 8th graders (age range of approximately 6 to 14 years) and college students with an old/new recognition task. They found no ORE in 6- to 8-year-olds. Only older children, from 9 years on, and adults showed the ORE.

A similar pattern of results was found by Goodman et al. (2007). They tested 5- to 13-year-old Caucasian children and adults from the US, Norway, and South Africa and biracial (Caucasian-African American) children and adults with Caucasian, African, and Asian faces. Participants were shown the target pictures for 2 s and were then asked to judge the profession of the person depicted. A recognition test was administered 2 days later. Irrespective of their nationality or experience with other-race faces 5- to 7-year-old children had equal recognition performance for all face types, whereas older children (from 8 years on) and adults showed the ORE. Goodman et al. (2007) discussed experimental demands as one factor likely causing these differences between the age groups. They claimed that the younger children in their study possibly spent attentional resources searching for a cue about the person’s profession and were, therefore, not focusing enough on the face recognition task.

All in all, comparing the studies that found the ORE in childhood with those that did not find the ORE, it can be assumed that the inconsistencies in results are probably due to methodological differences between the studies. Thus, from studies on the ORE in older children it is difficult to draw a hypothesis on the transition of the ORE from infancy to children at 3 years of age.

Therefore, in the current experiments^1^, we wanted to further investigate the ORE in 3-year-old children. We intended to expand previous studies by examining 3-year-olds from two different ethnic groups, Caucasian, that is German children, and African, that is Cameroonian children with the same face material, Caucasian and African faces. Based on infant studies indicating the ORE in different ethnic groups we expected to find the ORE in Caucasian and African children.

Children were presented with either Caucasian or African female faces in a forced choice paradigm and a presentation time of 3 and 5 s in Experiment 1 (German children) and Experiment 2 (Cameroonian children), respectively, was used. We decided to extend the presentation times because Sangrigoli and de Schonen (2004) induced the ORE in the 3-year-olds by prolonging presentation times. We wanted to ensure that the children had enough time for encoding the faces and we intended to prevent floor effects in general. Since presenting the target faces longer mainly improved own-race recognition in Sangrigoli and de Schonen (2004) it was our assumption that longer presentation times would facilitate the emergence of experience related recognition advantages of faces in both groups. This means a higher recognition performance for African faces in Cameroonian children and for Caucasian faces in German children.

## EXPERIMENT 1

### METHODS

#### Participants

Participants were 41 Caucasian children living in Germany at the age of 3 years (mean age: 3 years 4 months, range 3.4–3.6, 17 male). These children were part of a larger group of children that had participated in several learning and memory tasks when they were infants. As we used different methods in the previous infant studies compared to the present study we did not expect any transfer effects. Children were randomly assigned to the conditions described beneath. Parental consent was obtained. Five additional children were tested but were excluded from the analyses because they did not understand the instructions (2 children), or were not keeping up with the task from the beginning (3 children).

#### Stimuli

**FIGURE 1:**
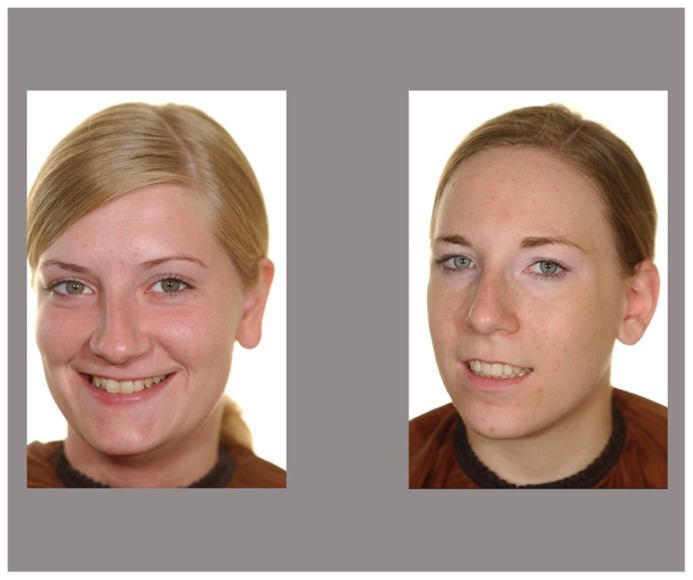
**Example of a test slide in the Caucasian face condition.** Displayed are a target and a distractor face shifted towards the same side.

**FIGURE 2:**
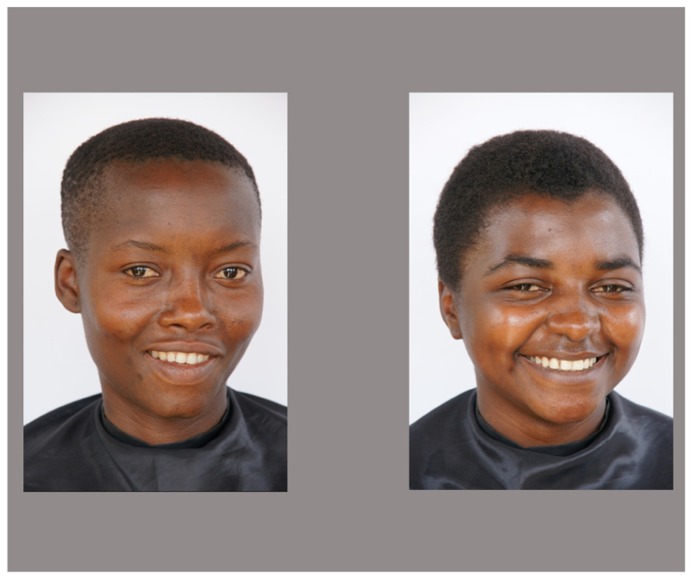
**Example of a test slide in the African face condition**. Test slides depict a target and a distractor face shifted towards the same side.

Stimuli comprised a set of color photographs of faces of 15 Caucasian and 15 African women of the approximate same age range (aged 20–34 years). None of them were familiar to the children. There were two pictures of each person, one in frontal view and a second one with a shift of the head of approximately 15° toward left or right, all of them were smiling and looking directly into the camera. Photographs were taken against a neutral white background with all women wearing a cape to eliminate cues from clothing and none of them wearing jewelry or glasses. In the experiment, faces were presented in white rectangles on a light gray background. Picture size was 13 cm × 19 cm. Each face served once as a target and once as a distractor, but not in the immediately following trial. Examples of faces pictures that we used are shown in **Figures 1** and **2**.

#### Procedure

Children were tested individually in a quiet room and were seated at a distance of approximately 60 cm in front of a 20 inch monitor (resolution 1600 × 900). E-Prime 2.0 software (Psychology Software Tools, Sharpsburg, PA, USA) was used to present the stimuli and to record children’s responses and reaction times. The experimenter was sitting next to the child and started the presentation of each trial separately when the child attended to the screen and entered children’s responses. 

Participants were randomly assigned to the condition in which Caucasian faces or to the condition in which African faces were presented. Each session started with learning trials including a set of butterfly pictures during which the forced choice recognition task was introduced. Children were presented with a picture of a butterfly presented in the middle of the screen for 3 s. After an inter stimulus interval of 2 s the target butterfly was presented paired with a picture of a different butterfly. Children then had to decide which one of the two butterflies they had seen before. After that, the actual face recognition task started. 

A target face was presented in frontal view positioned in the middle of the screen for 3 s. After an inter stimulus interval of 2 s the test slide was presented, displaying the target face and one distractor face side by side, 13 cm apart. Both faces on the test slide were shown in the same pose, and shifted toward the same side (left or right for half of all trials). Children then had to decide which of the two faces was presented before. To ensure that children were attentive to both faces on the test slide the experimenter made a smooth hand movement from the bottom left to the right lower corner of the screen. The first three trials served as practice trials, where the experimenter gave feedback and instructed children to look carefully at the two different women. After that, 15 test trials, divided into two blocks of eight and seven trials, started. No feedback was given during the test trials. To keep children focused on the task one of two different attention-getters (comic picture accompanied by a sound) was presented in the middle of each block. The blocks themselves were separated by a short break.

### RESULTS

Trials where children took longer than 30 s to respond were excluded completely from further analyses to remove unusually slow responses, e.g., when children were not paying attention (3 trials in Caucasian condition and 3 trials in African condition). Mean recognition accuracy as percentage of correct trials over all test trials was calculated for each child within each condition. All scores of the test trials were included in the analyses independent of the results during the three practice trials where feedback was given. Since sex has been shown to play a role in face recognition accuracy (Rehnman and Herlitz, 2006) it was included in the analyses in addition to ethnicity.

#### Accuracy

A two-way ANOVA with face ethnicity (African or Caucasian) and sex (male or female) on children’s mean recognition accuracy was carried out. Analyses revealed a significant main effect of ethnicity, *F*(1,37) = 5.47, *p* < 0.05, $\eta_{p}^{2}$ = 0.13, with Caucasian faces being recognized more accurately (*M *= 72.8%, *SD* = 16.89) than African faces (*M* = 62.1%, *SD* = 14.21), and a main effect of sex, *F*(1,37) = 6.72, *p* < 0.05, $\eta_{p}^{2}$ = 0.15, with girls, (*M* = 73.3%, *SD* = 14.98) outperforming boys, (*M* = 61.3%, *SD* = 16.46), but no interaction between ethnicity and sex, *F*(1,37) = 1.01, *p* > 0.05. Two-tailed *t*-tests showed that performance in both conditions differed significantly from chance level (50% accuracy), *t*(23) = 6.61, *p* < 0.001; *t*(16) = 3.50, *p* < 0.01, for Caucasian and African faces respectively.

### DISCUSSION

The results showed a clear advantage for own-race faces in 3-year-olds’ recognition accuracy. Moreover girls outperformed boys. The demonstration of higher accuracy for own-race faces confirms the findings of Sangrigoli and de Schonen (2004), and extends them to a different ethnic group. The main effect of sex was not the focus of this study. Nevertheless, it fits in with previous research demonstrating better performances of female participants in face recognition tasks for adults (Sommer et al., 2013), as well as for children aged 7 to 9 years (Rehnman and Herlitz, 2006), especially when female faces were presented as stimuli. Attention to female faces by female participants is discussed as one factor driving the sex differences (Herlitz et al., 2008; Loven et al., 2011), which have previously not been shown at that early age.

## EXPERIMENT 2

In Experiment 2 we aimed at investigating whether 3-year-old Cameroonian children show a corresponding ORE as the German 3-year-olds in Experiment 1 when the same face stimuli were used. As the Cameroonian children’s daily life differs in many respects from that of German children we had to modify the procedure of the experiment in order to compensate for those differences. Cameroonian children from our sample comprised children from the Nso tribe who possessed little experience with photographs of people in their daily life. Questionnaires showed that only a minority of their families possessed a TV or computer so that the children also had less experience with pictures displayed on a screen in general, compared to German children. Therefore presentation times of the stimuli were extended to 5 s and children were given extra practice trials in a training session to get familiar and comfortable with the task described in Experiment 1.

### METHODS

#### Participants

Participants were 66 Cameroonian children at the age of 3 years (mean 3 years 4 months, range 3.3–3.5, 32 male) from the Nso tribe living in a rural area in the Bamenda Grassfield in Cameroon. Children were tested at a local health center. As in Experiment 1, children were part of a larger group of children that had participated in several learning and memory tasks when they were infants. As we used different methods in the previous infant studies compared to the present study we did not expect any transfer effects. Children were randomly assigned to the conditions described beneath. Parental consent was obtained. Seven additional children were tested but were excluded from analyses because they pointed to only one side of the screen throughout the whole experiment (5 children), due to experimental error (1 child) or poor health (1 child).

#### Stimuli

For the face recognition test a subset comprising pictures of 12 African and 12 Caucasian female faces out of the 15 faces from Experiment 1 was used. None of them were familiar to the children. In order to facilitate the task compared to the task of Experiment 1, half of the target faces were presented in frontal poses both during presentation and test while for the other half of the target faces, test faces were slightly varied to left or right for test, as in Experiment 1.

In addition to the photos of faces we used three pairs of real objects (block, shoe lace, spoon, comb, cup, pencil) and pictures of these objects as well as pictures of 12 other objects (e.g. shoe, plate, button, ball) from the Bank of Standardized Stimuli (BOSS; Brodeur et al., 2010) in a training session.

#### Procedure

Children were tested individually in a quiet room and were seated at a distance of approximately 60 cm in front of a 20 inch monitor (resolution 1680 × 1050). E-Prime 2.0 software (Psychology Software Tools, Sharpsburg, PA, USA) was used to present the stimuli and to record children’s responses and reaction times. A native experimenter sat next to the child and led the child through the experiment. When children attended to the screen a second experimenter from Germany positioned outside of the child’s focus started the presentation of each trial and entered children’s responses.

Children were presented with two sessions. The first session was considered a training session before, in a second session, the actual face recognition task was administered. The purpose of the training session was to allow the children to get familiar to the computerized forced choice recognition task and to the use of photographic material. In the training session, first of all real objects were used as stimuli, and after that, pictures of the same objects were used which were presented on a computer screen. One object (spoon, pencil, shoe lace, block, cup or comb) was shown to the children and after a short moment the same object and a second object were presented to the children simultaneously and they were asked to identify the object seen before. After that the computer presentation was introduced by showing pictures of the same objects on the screen. Children saw a target picture (object) for 5 s, followed by a blank screen for 2 s and then the target picture and a distractor picture were presented simultaneously and children were asked to point to the target seen before. After those training trials, in which we used objects as stimuli, another training session with faces as stimuli followed. We presented 12 trials, where children had to either distinguish African faces from Caucasian faces or faces of the two ethnicities from pictures of objects. The faces used differed from those used in the actual face recognition task.

The actual face recognition task, where children were presented with either Caucasian or African faces, was administered at another day. Children were randomly assigned to either the Caucasian or the African condition. The face recognition task started with three forced choice recognition trials with pictures of the objects used in the training session. Thereafter, faces were introduced as stimuli. During the first three trials the experimenter gave feedback and instructed the children to look carefully at the two different women. After that, 12 face recognition trials, divided into two blocks of four and eight trials, started. During the trials no feedback was given. To keep children focused on the task one of two different attention-getters (comic picture accompanied by a sound) was presented between the blocks.

### RESULTS

When reaction times exceeded 30 s trials were excluded from analyses to remove unusually slow responses (1 trial within African condition). Mean recognition accuracy as percentage of trials with correct responses over all 12 test trials was calculated for each child within each condition. All scores of the test trials were included in the analyses independent of the results during the three practice trials where feedback was given. Since sex had an effect on accuracy in Experiment 1 it was included in the analyses here as well.

#### Accuracy

A two-way ANOVA with face ethnicity (African or Caucasian) and sex (male or female) on recognition accuracy was carried out. A significant main effect of ethnicity, *F*(1,62) = 5.67*, p* < 0.05, $\eta_{p}^{2}$ = 0.08, with Caucasian faces being recognized at a lower level (*M* = 53.8%, *SD* = 14.6) than African faces (*M* = 62.3%, *SD* = 15.09), was found. The accuracy for African faces differed significantly from chance level, with *t*(32) = 12.26, *p* < 0.001, whereas Caucasian faces were recognized at chance level, *t*(32) = 3.79, *p* > 0.05. No main effect of sex or interaction between sex and ethnicity, has been revealed, both *p’*s > 0.05.

## COMPARISON BETWEEN EXPERIMENT 1 AND 2

**FIGURE 3:**
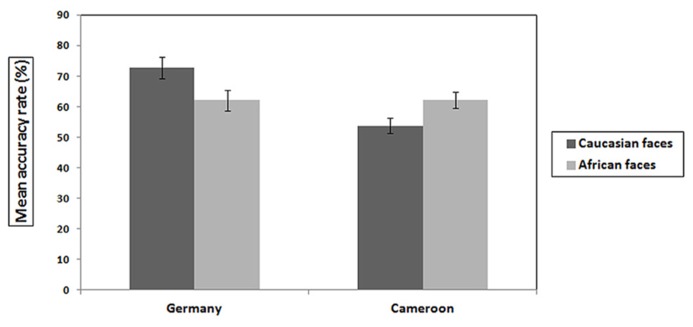
**Accuracies over all test trials for German (left) and Cameroonian children (right) for Caucasian and African faces**.

Comparing the accuracy results from the two experiments with a three-way ANOVA including the factors cultural background (German or Cameroonian children), face ethnicity (African or Caucasian faces), and sex (male or female) showed a main effect of cultural background, *F*(1,99) = 7.36, *p* < 0.05, $\eta_{p}^{2}$ = 0.07, with higher accuracies in the German sample (*M* = 68.4%, *SD* = 16.53) than in the Cameroonian sample (*M* = 58%, *SD* = 15.34) and an interaction of cultural background and ethnicity, *F*(1,99) = 10.82, *p* < 0.05, $\eta_{p}^{2}$ = 0.10, with higher accuracies for Caucasian faces in German children and for African faces in Cameroonian children (see Experiments 1 and 2 for details, compare also **Figure 3**). Also, an interaction was found between cultural background and sex, *F*(1,99) = 5.18, *p* < 0.05, $\eta_{p}^{2}$ = 0.05, with girls outperforming boys in the German sample only. No main effects of sex or ethnicity were found, and the two-way interaction between these two factors, as well as the three-way interaction with cultural background were not significant, all *p’*s > 0.05.

### DISCUSSION

The results presented here showed an advantage for the recognition of own-race faces in Cameroonian children. Cameroonian children were more accurate for faces of their own ethnic group than for faces of a different ethnic group. The sex differences found in the German sample were not present in the Cameroonian group. Girls and boys showed equal abilities to recognize individual faces. Comparing the performance of the German and Cameroonian samples revealed the expected interaction between cultural background and ethnicity in accuracies, own-race faces were recognized more accurately in both samples. Both groups showed a clear ORE. Despite these analogous results, German children were more accurate than Cameroonian children, above chance even when recognizing African faces even though they had target presentation times of only 3000 ms compared to 5000 ms in the Cameroonian group. As both experiments analyzed the same dependent variables and used the same stimuli in a matching paradigm it seems justified to compare their results. However, at the same time both experiments differed in some aspects of the experimental procedures. Therefore, the interpretation of the results of such a direct comparison between the experiments has to be made with precaution.

## GENERAL DISCUSSION

The main focus of this study was to investigate the ORE in 3-year-old children from two different cultural backgrounds, Germany and Cameroon, using the same face material to learn more about the transition of the ORE from infancy into childhood. Being presented with either Caucasian or African faces, 3-year-olds from both cultural backgrounds showed greater recognition accuracy for own-race faces compared to other-race faces.

Our findings are in accordance with studies showing that the ORE is present in children beyond infancy (Pezdek et al., 2003; Sangrigoli and de Schonen, 2004; De Heering et al., 2010) and contradicts those that questioned the effect in older children (Chance et al., 1982; Goodman et al., 2007). In particular, our results are closely related to the ones by Sangrigoli and de Schonen (2004). In a similar paradigm using pictures of Caucasian and Asian faces, they found the ORE in Caucasian children as young as 3 years when children had sufficient encoding time. In our study, with 3000 and 5000 ms of presentation time for the German and Cameroonian children, we used considerably longer presentation times than Sangrigoli and de Schonen in order to ensure that the 3-year-olds were indeed provided with enough time to encode the face stimuli and to prevent floor effects. As both Cameroonian and German children performed above chance level for own-race faces we conclude that the presentation times in fact allowed for sufficient encoding of the faces.

With the affirmative results on the ORE from early and middle childhood and the infant results on the emergence of the ORE, our results suggest that the ORE continues into early childhood. Once developed it appears to be a stable phenomenon in face recognition that is present from infancy on, despite the shift in paradigm between infancy and childhood, when looking time measures are replaced by more active verbal or motor measures of child behavior. Given the results on discrepancies between infant and childhood measures from other domains of cognitive development, in face recognition this transition seems to pose less of a challenge than in, for example, studies on intuitive physics (Aschersleben et al., 2013). The reasons for that could be seen in differences between the domains of cognitive competencies themselves and in the corresponding task demands. It can be assumed that it is easier to memorize a face than to apply intuitive knowledge on physical events. Similarly, it seems as if the task shifts from infancy to early childhood are smaller in the domain of face recognition than in the domain of intuitive physics. In infants, face recognition is measured in terms of looking times whereas in young children, recognition is measured by pointing or verbal responses when the target faces is paired with a distractor face. No further knowledge has to be applied to choose the correct answer. Regarding intuitive physics, infants’ understanding of physical events is measured by looking times when unexpected or impossible events are presented. Young children, however, usually are confronted with cognitively more demanding questions about a physical event and also have to actively apply knowledge on physical events they have acquired outside the experiment.

In contrast to the findings above, the studies by Chance et al. (1982) and Goodman et al. (2007) did not find the ORE in their youngest age groups, 5- to 7-, and 6- to 8-year-olds, respectively. What distinguishes these studies from our study and also from the one by Sangrigoli and de Schonen (2004), aside from different age groups and national backgrounds, is the method being used. Chance et al. (1982) asked participants to look at 16 target pictures for 5 s successively and then presented them with an old/ new recognition task right after, instead of using a matching paradigm as we did. They found an ORE in all but the youngest age group. They argued that children’s performance was above chance level and indicated in fact no ORE. Goodman et al. (2007), who found similar results, claimed that their own task, to make job decisions during encoding of the face pictures, might have distracted the youngest ones from the actual face recognition task, which could be responsible for not showing the ORE. Further research is needed to clarify whether the differing results of those studies and our studies are in fact due to the differences of the methods used.

Our results extend previous findings by demonstrating, first, that the ORE is also present in 3-year-olds, when pictures from a different ethnic group are used (African faces in contrast to Asian faces as in Sangrigoli and de Schonen, 2004). This parallels findings from infant studies (Kelly et al., 2009) and also adult studies (Meissner and Brigham, 2001) showing the ORE for faces from various ethnic groups. Thus, it is not that faces of one particular other ethnic group are harder to distinguish. It is rather experience with faces from one’s own group that drives better recognition of own-race faces (Bar-Haim et al., 2006; De Heering et al., 2010). Second, our results add to previous research by demonstrating an ORE in German and Cameroonian children with the same stimulus material, the same stimuli served alternatively as own- and other-race faces for each of the two groups. While most studies investigated the ORE from just one side, our approach rules out stimulus based explanations for the observed difference between own- and other-race faces. Since we find the effect in both groups, differences in level of difficulty between the stimulus sets cannot be driving the results.

Even though the ORE was comparable in size in both samples, we found significantly higher accuracies within the German sample compared to the Cameroonian children. But, does that mean that the development of face recognition is protracted in Cameroonian children or that their face recognition performance is generally at a lower level? We think this performance difference does not reflect actual differences in face recognition abilities between the two cultures. We assume that the difference can be due to the fact that the general demands of the face recognition task fit better to the daily life experiences of the German children than to those of the Cameroonian children. German children possess more media experience and photographic material is always present in their daily life whereas the Cameroonian children stem from a rural area where most families do not possess a TV and where photographic material is rather an exception. Regardless of our effort to customize the task for the Cameroonian children by offering an extra training session and also longer presentation times, this may not have been sufficient and the main reason for the obtained level differences in face recognition.

Future studies that either use tasks that build more strongly on common knowledge, e.g. in the media used, or provide more training could help to settle this issue. Moreover, the fact, that both groups display a comparable ORE at age three allows no conclusions about the developmental trajectory in the two groups. Longitudinal research that also takes into account methodological issues as mentioned above starting in the first year of life into childhood is needed to investigate whether the ORE develops at the same rate in different ethnic groups.

To conclude, the present study aimed at investigating the ORE in early childhood by using the same stimulus set in children from Germany and Cameroon and found the ORE in both groups at 3 years of age. This indicates that the ORE emerging during infancy is present in early childhood in children from different cultural backgrounds and is shown under certain, facilitating experimental conditions.

## Conflict of Interest Statement

The authors declare that the research was conducted in the absence of any commercial or financial relationships that could be construed as a potential conflict of interest.

## Footnotes

1 All research has been realized in accordance with the Ethical Principles of Psychologists and the Code of Conduct of the American Psychological Association.
